# Describing Uncertainty in *Salmonella* Thermal Inactivation Using Bayesian Statistical Modeling

**DOI:** 10.3389/fmicb.2019.02239

**Published:** 2019-09-25

**Authors:** Kento Koyama, Zafiro Aspridou, Shige Koseki, Konstantinos Koutsoumanis

**Affiliations:** ^1^Laboratory of Food Microbiology and Hygiene, Department of Food Science and Technology, School of Agriculture, Forestry and Natural Environment, Aristotle University of Thessaloniki, Thessaloniki, Greece; ^2^Graduate School of Agricultural Science, Hokkaido University, Sapporo, Japan

**Keywords:** uncertainty, predictive microbiology, Bayesian, bacterial inactivation, global regression model

## Abstract

Uncertainty analysis is the process of identifying limitations in scientific knowledge and evaluating their implications for scientific conclusions. In the context of microbial risk assessment, the uncertainty in the predicted microbial behavior can be an important component of the overall uncertainty. Conventional deterministic modeling approaches which provide point estimates of the pathogen’s levels cannot quantify the uncertainty around the predictions. The objective of this study was to use Bayesian statistical modeling for describing uncertainty in predicted microbial thermal inactivation of *Salmonella enterica* Typhimurium DT104. A set of thermal inactivation data in broth with water activity adjusted to 0.75 at 9 different temperature conditions obtained from the ComBase database (www.combase.cc) was used. A log-linear microbial inactivation was used as a primary model while for secondary modeling, a linear relation between the logarithm of inactivation rate and temperature was assumed. For comparison, data were fitted with a two-step and a global Bayesian regression. Posterior distributions of model’s parameters were used to predict *Salmonella* thermal inactivation. The combination of the joint posterior distributions of model’s parameters allowed the prediction of cell density over time, total reduction time and inactivation rate as probability distributions at different time and temperature conditions. For example, for the time required to eliminate a *Salmonella* population of about 10^7^ CFU/ml at 65°C, the model predicted a time distribution with a median of 0.40 min and 5th and 95th percentiles of 0.24 and 0.60 min, respectively. The validation of the model showed that it can describe successfully uncertainty in predicted thermal inactivation with most observed data being within the 95% prediction intervals of the model. The global regression approach resulted in less uncertain predictions compared to the two-step regression. The developed model could be used to quantify uncertainty in thermal inactivation in risk-based processing design as well as in risk assessment studies.

## Introduction

Food safety management has been changing to a more risk-based approach for food safety control with regulators all over the world adopting microbial risk assessment as the foundation for their decision-making (Koutsoumanis and Aspridou, 2016). The dynamics of pathogens’ growth, survival, and inactivation in foods should be explicitly considered in microbiological risk assessment. Predictive microbiology has been recognized as an important part of the risk assessment for estimating the changes in microbial numbers in foods along the farm-to-fork chain (Kakagianni and Koutsoumanis, 2019). However, the application of predictive models in risk assessment has different demands compared with “traditional” predictive microbiology (Ross and McMeekin, 2003). The Codex guidelines for conducting microbiological risk assessment apparently stress the importance of the concept of uncertainty and variability (CAC, 1999). In risk-based approaches, the use of deterministic models which provide point estimation of microbial concentrations is problematic (Nauta, 2002) and the need for development of the models which are capable of expressing populations of pathogens probabilistically (i.e., predict the probability distribution of the microbial numbers at the time of consumption) has been emphasized (Nauta, 2002; Ross and McMeekin, 2003; Koutsoumanis et al., 2016).

In the context of risk assessment, uncertainty is used as a general term which is relevant to all types of limitations in the available knowledge that influences the range and probability of possible answers to the assessment question (EFSA Scientific Committee et al., 2018). Risk assessors should examine in a systematic way every part of their assessment in order to identify and characterize all uncertainties, including those related with the inputs to the assessment as well as the methods and models used in the assessment. In microbial risk assessment, the uncertainty in the predicted microbial behavior can be an important component of the overall uncertainty. Indeed, the description of the microbial behavior in exposure assessment is uncertain with the most important sources of uncertainty related to the experimental error of the data used for the development of the model as well as the fitting errors of the models (Nauta, 2002; Pouillot et al., 2003).

One approach for quantifying uncertainty in predictive microbiology is the Bayesian inference (Carlin and Louis, 2000; Congdon, 2007) where all the model parameters are random variates and not fixed as other frequentist statistical approaches. In a Bayesian framework, prior distributions for all model parameters which reflect the state of knowledge available before analyzing the data set are specified. In a second step, posterior distributions for all parameters are computed using Bayes’ theorem by combining prior distributions and observed data (Pouillot et al., 2003). Bayesian inference has been used for estimation of model parameter uncertainty in several microbial risk assessment studies (Pouillot et al., 2003; Barker et al., 2005; Crepet et al., 2006; Delignette-Muller et al., 2006; Crépet et al., 2009). In most of these studies, uncertainty is characterized for microbial growth while less information are available for the applicability of Bayesian inference in microbial inactivation models and the interpretation of parameter uncertainty for describing reduction times for a microbial population (Membré and van Zuijlen, 2011).

Apart from the experimental errors another important uncertainty source in microbial behavior, predictions is the errors in fitting the data to the models. Predictive microbiology models are usually fitted to observed data in a two-step fitting process. In the first step, the primary growth model is fitted to experimental data and the kinetic parameters are estimated. The second step is to independently fit a secondary model to each of these kinetic parameters as a function of experimental factors (e.g., temperature, pH, water activity, etc). The global fitting is an alternative procedure in which primary and secondary models are combined, which provides a direct relationship between environmental factors and microbial counts. The selection of the above fitting procedures can significantly affect the uncertainty in model predictions (Martino and Marks, 2007).

The objective of this study was to use Bayesian statistical modeling for describing uncertainty in predicted microbial thermal inactivation of *Salmonella enterica* Typhimurium DT104 by comparing a two-step and a global Bayesian regression. Such a model can provide predictions as probability distributions which enables to quantify uncertainty related to model fitting in risk-based processing design and in microbial risk assessment studies.

## Materials and Methods

### Data

A set of *Salmonella enterica* Typhimurium DT104 thermal inactivation data at 9 different temperature conditions (55, 60, 65, 70, 72, 74, 76, 78, and 80°C) obtained from the ComBase database^1^ was used for the development and validation of the models. Data were reported by Mattick et al. (2001) who investigated the bacterial inactivation of the pathogen in the broth adjusted to water activity value of 0.75 by glucose-fructose. Dilutions were made in maximal recovery diluent, and viable counts were estimated with plating onto blood agar and incubation for 48 h at 37°C to ensure optimal recovery of injured cells. An initial cell density of approximately 10^7^ CFU/ml was used. Three to four replicates were tested at each temperature (32 inactivation curves in total). Data at 8 temperature conditions (55, 60, 65, 70, 74, 76, 78, and 80°C) were used for model development and one temperature (72°C) for model validation.

### Bayesian Modeling

#### Global Regression

In global Bayesian regression the primary model used to describe the concentration of survivors over time was combined with the secondary model describing the relation between inactivation rate and temperature. As a primary model a log-linear model was used (Peleg, 2006):

(1) $${\text{log}}_{10}\,N_{t}=-k\,\left(T\right)\,t+{\text{log}}_{10}\,N_{0}$$

where *N*_*0*_ [log_10_ CFU/ml] is the initial population size at time 0 [h], *t* is the inactivation time [h], *k*(*T*) is temperature-dependent inactivation rate parameter [log_10_ CFU/h] at temperature *T* [°C]. For *N*_*0*_ the mean value of all observed initial cell densities was used. The random variable of inactivation rate parameter was described as a log-normal distribution:

(2) k⁢(T)∼LogNormal⁢(log⁡(μ),σ)

where μ and σ are parameters of log-normal distribution. An exponential model was used to describe the inactivation rate parameter μ as a function of temperature as following:

(3) μ=b×exp⁢(c×T)

where *b* and *c* are scaling parameter and exponential rate parameter respectively. In global Bayesian regression Eqs. (1)–(3) were combined as following.

(4) $$\frac{1}{t}\,{\text{log}}_{10}\,\frac{N_{0}}{N_{t}}∼\text{LogNormal}\,\left(\log\left(b\times \text{exp}\,\left(c\times T\right)\right),\sigma \right)$$

Eq. (4) was fitted to the observed *Salmonella* inactivation data at 55, 60, 65, 70, 74, 76, 78, and 80°C to obtain posterior distributions for *b*, *c*, and *σ* parameters.

#### Two-Step Regression

For comparison, bacterial inactivation was also modeled with a two-step approach using Bayesian regression. At first, the primary model was fitted independently to the data at each temperature (*T*_sep_ = 55, 60, 65, 70, 74, 76, 78, and 80°C).

(5) $${\text{log}}_{10}\,N_{t}=-k_{T_{\text{sep}}}\,t+{\text{log}}_{10}\,N_{0}$$

Then, the estimated inactivation rates *k*_*T*_sep__ were described as log-normal distributions:

(6) $$k_{T_{\text{sep}}}∼\text{LogNormal}\,\left(\text{log}\,\left(\mu_{T_{\text{sep}}}\right),\sigma_{T_{\text{sep}}}\right)$$

where *μ*_*T*_sep__, *σ*_*T*_sep__ are parameters estimated from the primary model fitting. We can obtain the uncertainty of *k*_*T*_sep__ by using joint posterior distribution *μ*_*T*_sep__ and *σ*_*T*_sep__. 100 values for each *k*_*T*_sep__ were selected randomly from the distribution to build a data set of inactivation rates at temperature *T*_sep_.

(7) $$k_{\text{two}}\,\left(T\right)=\left\{k_{55\,i},k_{57\,i},\ldots ,k_{T_{\text{sep}}\,i},\ldots ,k_{80\,i}\right\},i=1,2,3,\ldots ,100$$

where *k*_*T*_sep_*i*_ and *k*_two_(*T*) are *i*th random sampling from *k*_*T*_sep__ and the data set of inactivation rates obtained from the primary fitting at all temperatures *T*_sep_. The data set of inactivation rates *k*_two_(*T*) were fitted to the secondary model as following:

(8) $$k_{\text{two}}\,\left(T\right)∼\text{LogNormal}\,\left(\text{log}\,\left(\mu_{\text{two}}\right),\sigma_{\text{two}}\right)$$

(9) $$\mu_{\text{two}}=b_{\text{two}}\times \text{exp}\,\left(c_{\text{two}}\times T\right)$$

*μ*_two_, *b*_two_ and *c*_two_ are mean values of inactivation rate, scaling parameter and exponential rate parameter respectively. By combining Eq. (8) and Eq. (9) the secondary model has the following form:

(10) $$k_{\text{two}}\,\left(T\right)∼\text{LogNormal}\,\left(\log\left(b_{\text{two}}\times \text{exp}\,\left(c_{\text{two}}\times T\right)\right),\sigma_{\text{two}}\right)$$

*Salmonella* inactivation data were fitted to Eq. (10) and the posterior distributions of *b*_two_, *c*_two_, and *σ*_two_ were obtained.

#### Computation

Bayesian inference can combine prior parameters into models. Prior distributions can be set by using previous knowledge on the parameters. It would be helpful when prior studies or some experiences are known. Prior distribution has been used in several studies related to bacterial growth or inactivation model (Pouillot et al., 2003; Crepet et al., 2006; Crépet et al., 2009; Spor et al., 2010; Jaloustre et al., 2011). Non-informative prior distribution is also possible, when there is not enough information of parameters. In this study, we used uniform distribution as a non-informative prior distribution since there was no prior information.

Computations were performed using Stan software^2^ and rstan package of R software^3^. For each model, inferences were conducted with 5×10^3^ iterations with 4 independent chains after an adaptation phase of 2.5×10^3^ iteration. Convergence was checked by both visually checking Markov Chain Monte Carlo (MCMC) chain traces and examining Gelman and Rubin convergence statistic. The R code used for the above calculation is outlined in Supplementary Material.

## Results and Discussion

**FIGURE 1:**
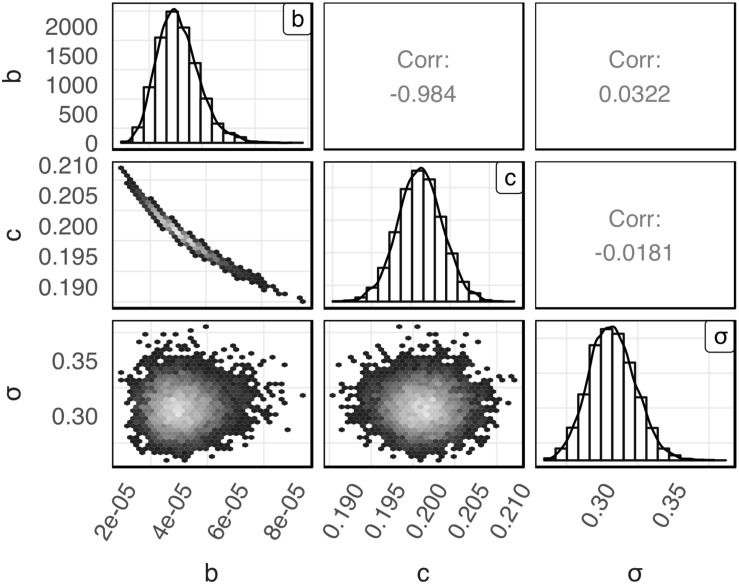
Empirical joint posterior distribution of parameters *b*, *c*, and σ (under the diagonal), corresponding adjusted distributions (on the diagonal) and correlation coefficients (over the diagonal) estimated with the global Bayesian regression.

The posterior distributions for model’s parameters *b*, *c*, and σ obtained with the global Bayesian regression are presented in Figure 1. The mean values for *b*, *c*, and σ were 4.10 × 10^–5^, 0.20 and 0.31, respectively. The respective coefficients of variation were 19.41, 1.42, and 5.29%. As shown in the latter figure the posterior distribution of *c* and σ were symmetric while the posterior distribution of *b* was slightly skewed to the right. Parameters *b* and *c* showed a high negative correlation (*r* = −0.984). A very low correlation was observed between parameters *b* and σ (*r* = 0.0322) and parameters *c* and σ (*r* = −0.0181).

**FIGURE 2:**
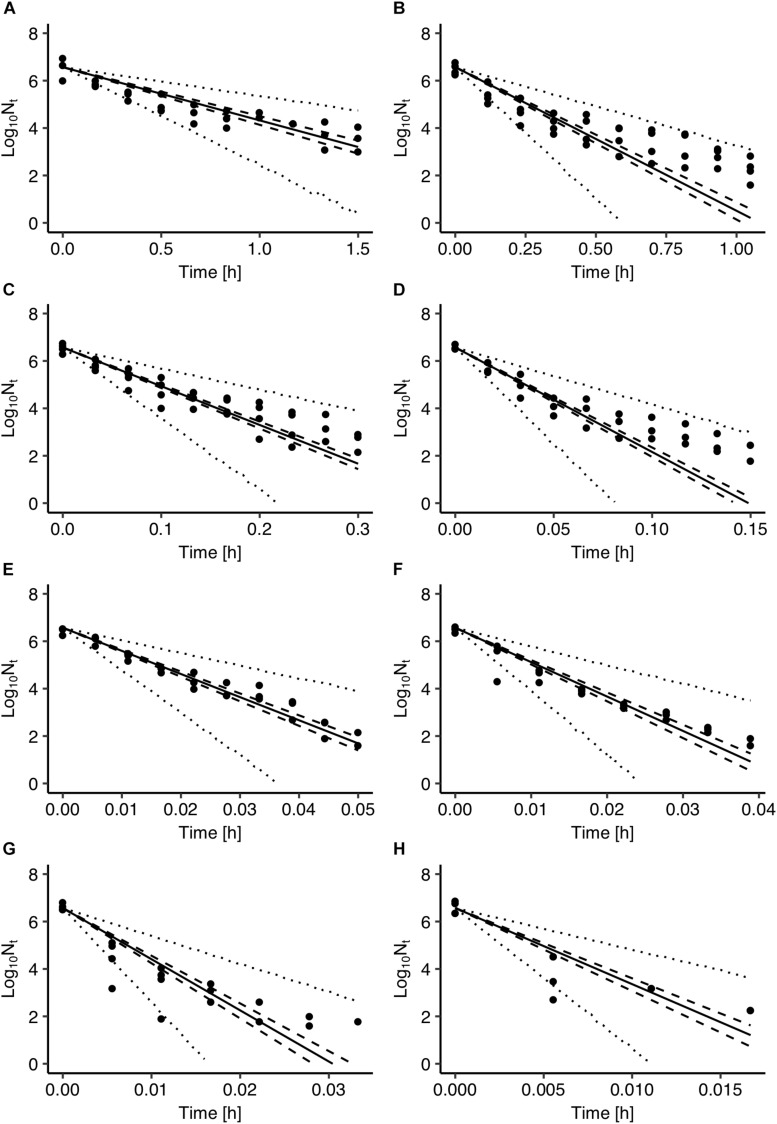
Comparison between observed cell densities with those estimated by the Salmonella inactivation model at 55°C **(A)**, 60°C **(B)**, 65°C **(C)**, 70°C **(D)**, 74°C **(E)**, 76°C **(F)**, 78°C **(G)**, and 80°C **(H)**. Solid lines represent prediction with parameters of maximum a posterior probability estimate. The 95% confidence intervals and prediction intervals are described with dashed and dotted lines, respectively.

Figure 2 presents a comparison between *Salmonella* inactivation predicted by the model and the observed data used for the development of the model. The results showed that the observed data are highly variable with the population range for some sampling times being up to 2 logs CFU/ml. In general, the observed variance in microbial behavior can be attributed to both variability and uncertainty (Nauta, 2002; Pouillot et al., 2003). In the case of microbial inactivation individual cell heterogeneity can be an important source of variability in population dynamics (Aspridou and Koutsoumanis, 2015). Aspridou and Koutsoumanis (2015) applied a statistical modeling approach for describing and evaluating the individual cell heterogeneity as variability source in microbial inactivation. Using Monte Carlo simulation, they showed that the variability in the predicted inactivation behavior is negligible for concentration above 100 cells due to the law of large numbers but as the concentration decreases below 100 cells the variability increases significantly. Considering however, that the data used in the present study were all above the threshold of 100 cells the variable behavior shown in Figure 2 can be mainly attributed to uncertainty and in particular to the experimental errors.

**FIGURE 3:**
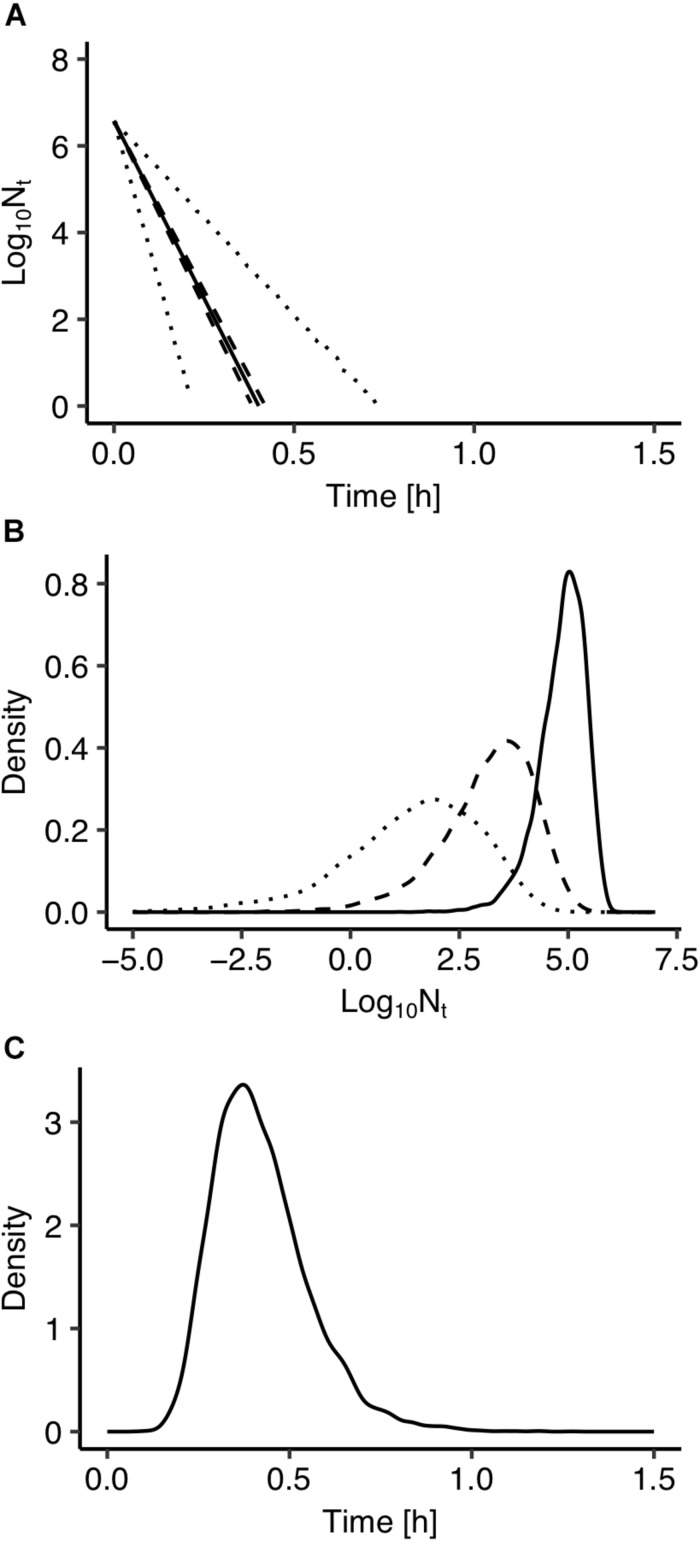
**(A)** Prediction of *Salmonella enterica* inactivation at 65°C. **(B)** Probability distributions describing uncertainty in the predicted cell density after 0.1 h (solid line), 0.2 h (dashed line), and 0.30 h (dotted line). **(C)** Probability distributions describing uncertainty in the predicted time for total inactivation of the population.

The use of Bayesian regression for the development of the model allowed for providing predictions in the form of probability distributions which characterize their uncertainty level. In Figure 2, solid lines represent the predictions made using the maximum posteriori probability estimate for the parameters of the model. The latter estimate is asymptotically equivalent to parameters estimated from maximum likelihood estimation (Robert, 2007). Dotted lines represent the confidence intervals and dashed lines represent the 95% prediction intervals based on the probability distributions of the model’s parameters presented in Figure 1. As shown in Figure 2, the majority of the observed data points are within the 95% prediction intervals. The description of the uncertainty by the developed model is more clearly demonstrated in Figure 3. Figure 3A presents the prediction of *Salmonella enterica* inactivation at 65°C with the respective confidence and 95% prediction intervals. For each temperature condition, the output of the model is a probability distribution describing the uncertainty around the prediction. Figure 3B shows the probability distributions of *Salmonella* cell density at 65°C after 0.1, 0.2, and 0.3 h. At 0.1 h, the median predicted value was 4.94 log CFU/ml and the 5th and 95th percentiles were 3.86 and 5.59 log CFU/ml. At 0.3 h, the probability distribution of the predicted cell density is wider due to the cumulative effect of inactivation rate uncertainty. In the latter case, the median predicted cell density was 1.63 log CFU/ml and the 5th and 95th percentiles were −1.59 and 3.61 log CFU/ml. Figure 3C presents the probability distributions describing uncertainty in the predicted time for the total inactivation of *Salmonella* population. The median predicted time was 0.40 min and the 5th and 95th percentiles were 0.24 and 0.60 min respectively. This information is of great importance for a risk-based food processing design supporting food business operators in decision making on the duration of thermal processing based on an accepted level of risk for pathogen’s survival (Koutsoumanis and Aspridou, 2016).

**FIGURE 4:**
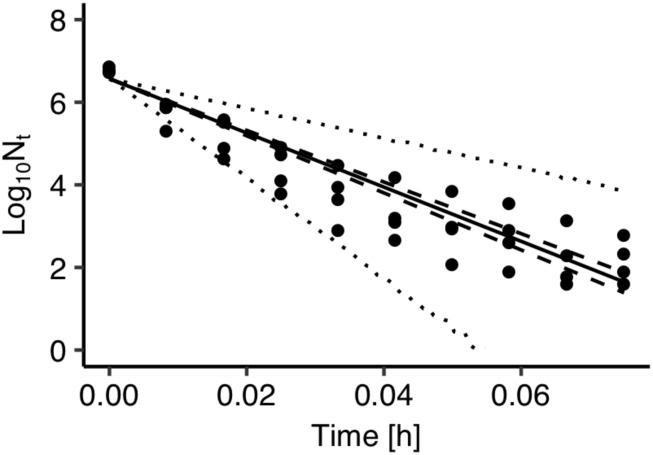
Comparison between observed (solid circles) and predicted inactivation of *Salmonella enterica* at 72°C. The solid, dashed and dotted lines represent the maximum *a posteriori* probability estimate (solid), 95% confidence band (dashed) of bacterial inactivation, and 95% prediction band (dotted) of bacterial inactivation.

The developed model was further validated against observed data on *Salmonella* thermal inactivation at 72°C. These data were not used for the development of the model. Figure 4 presents a comparison between the observed and the predicted inactivation. The model satisfactorily predicted the reduction of *Salmonella* population. The prediction based on the maximum posteriori probability estimate for the parameters of the model (solid line) was very close to observed data while most data points were within the 95% prediction intervals.

**FIGURE 5:**
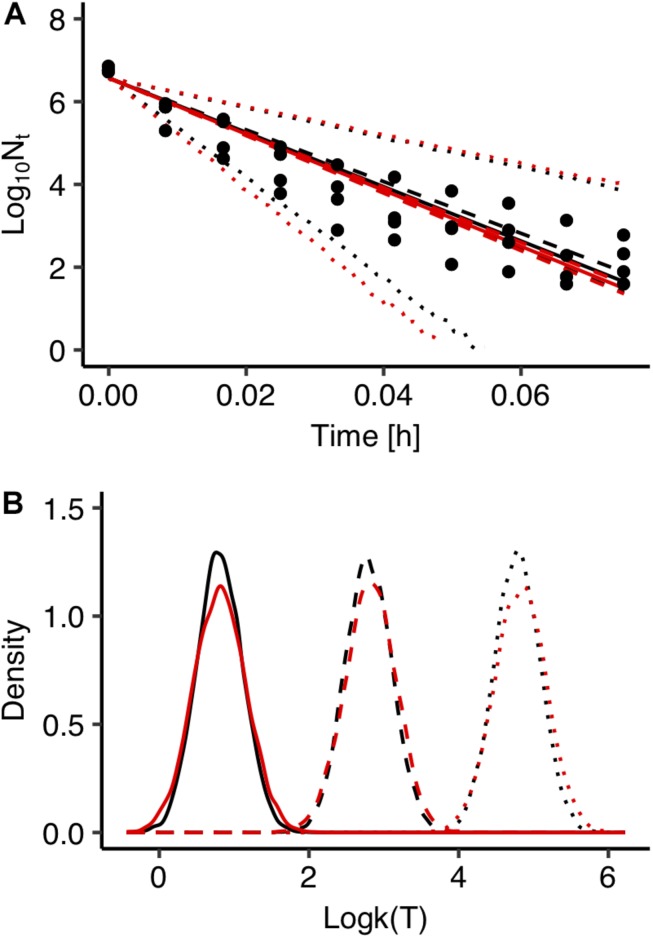
Comparison between global (black) and two step fitting (red) results. **(A)** Observed data (solid circles) and prediction of Salmonella enterica inactivation behavior under isothermal condition at 72°C. The solid, dashed and dotted lines represent the maximum a posterior probability estimate (solid), 95% confidence interval (dashed) and 95% prediction band (dotted). **(B)** Probability distributions of the estimated inactivation rate *log*⁡*k*(*T*) at 55°C (solid line), 60°C (dashed line), 65°C (dotted line).

Fitting errors are another important source of uncertainty in predictive microbiology. In most cases, predictive microbiology models are fitted to observed data in a two-step fitting process. In the first step, the primary model is fitted to the experimental data and the estimated kinetic parameters are further fitted to a secondary model. In general, there is no link between these two steps and uncertainty associated with the primary model is not taken into account in the secondary modeling. All kinetic parameters’ values of the primary models usually have the same weight in the secondary model regardless of the goodness of fit of the primary model (Pouillot et al., 2003; Martino and Marks, 2007). In order to evaluate the effect of the fitting process on the uncertainty of the model’s outputs, predictions of the model developed with the global fitting approach were compared with those derived from a two-step fitting method. For the two-step fitting, the data of *Salmonella* inactivation were first fitted to the primary model using Bayesian regression and the inactivation rate at each temperature was estimated in the form of probability distribution describing the uncertainty. At a second step, 100 values for each inactivation rate were selected randomly from the distributions and fitted to the secondary model. A comparison between the predictions of the model developed with the global and the two-step fitting method at 72°C is presented in Figure 5A together with the observed data. Figure 5B shows the probability distributions describing the uncertainty in the predicted inactivation rate at three temperature conditions. The comparison showed that the two-step approach results in a higher uncertainty of the model’s outputs compared with the global fitting. Previous studies comparing the two modeling procedures with maximum likelihood estimation also reported that the global approach provides less uncertain and more robust predictions. Martino and Marks (2007) compared global and two step fitting for a *Listeria monocytogenes* growth model and reported that the global regression yielded lower standard errors of calibration and it was more robust than the two-step procedure. Membré et al. (2004) combined primary and secondary models within a global approach that directly related the growth of *L. monocytogenes* to storage temperature and reported that the bias factor was significantly improved compared the two-step approach.

## Conclusion

In conclusion, the model developed in the present study using Bayesian regression enables to describe the uncertainty in predicted thermal inactivation of *Salmonella*. The model provides prediction in the form of probability distributions and can be used to quantify the uncertainty related to model fitting in risk-based processing design as well as in risk assessment studies. The model could be further improved by incorporating variability related to the heterogeneity in individual cell behavior (Aspridou and Koutsoumanis, 2015; Koyama et al., 2019). The Bayesian procedure could be also used to develop a complete model for *Salmonella* thermal inactivation enabling to describe variability and uncertainty separately.

## Data Availability Statement

Publicly available datasets were analyzed in this study. This data can be found here: www.combase.cc.

## Author Contributions

KeK, ZA, SK, and KoK designed the study and wrote the manuscript. KeK and ZA performed the study and analyzed the data.

## Conflict of Interest

The authors declare that the research was conducted in the absence of any commercial or financial relationships that could be construed as a potential conflict of interest.

## Footnotes

1 www.combase.cc

2 http://mc-stan.org

3 https://cran.r-project.org/web/packages/rstan/index.html
