# Supplementary figures and images for: Describing Uncertainty in Salmonella Thermal Inactivation Using Bayesian Statistical Modeling
Source: Front Microbiol. 2019 Sep 25;10:2239. doi: 10.3389/fmicb.2019.02239 (PMC6798057; doi:10.3389/fmicb.2019.02239)

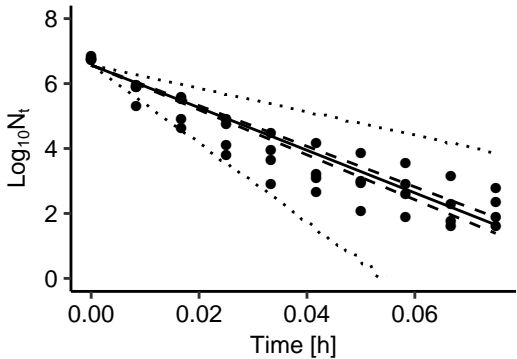

Supplement: Supplementary file 1 [file Data_Sheet_1.ZIP › bayesian_modeling/04_output/Fig.4.pdf]

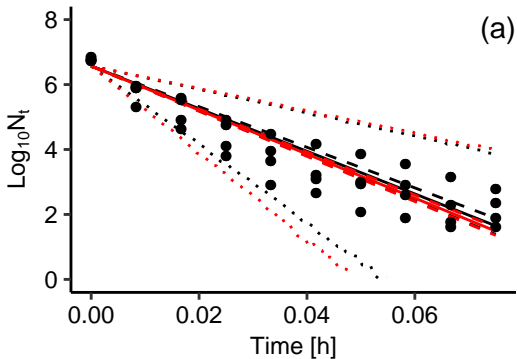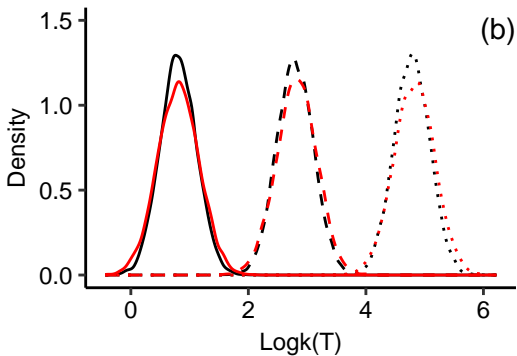

Supplement: Supplementary file 1 [file Data_Sheet_1.ZIP › bayesian_modeling/04_output/Fig.5.pdf]

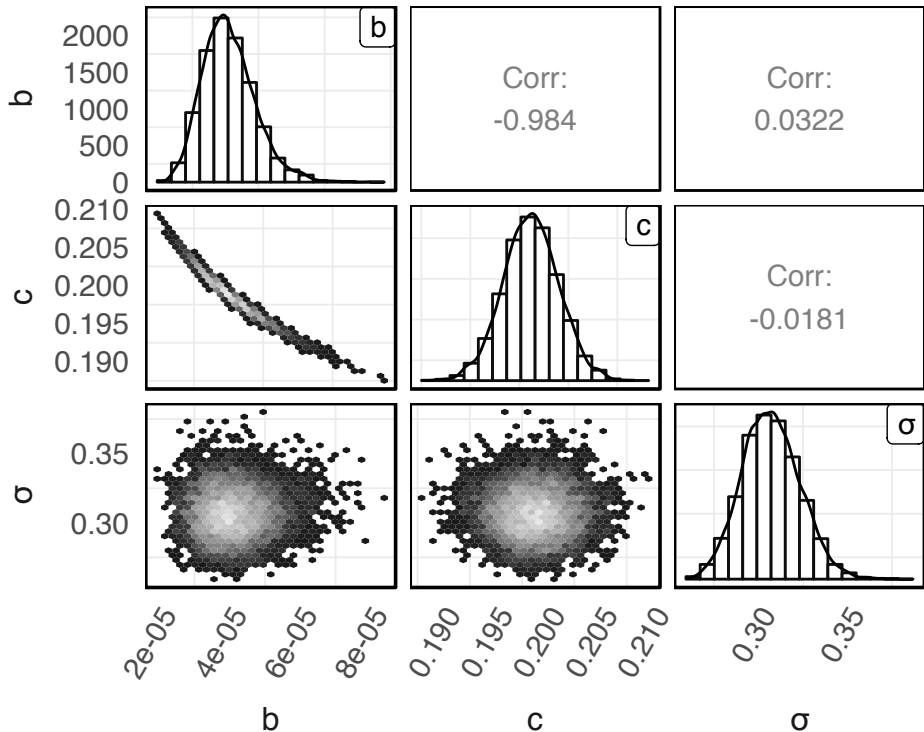

Supplement: Supplementary file 1 [file Data_Sheet_1.ZIP › bayesian_modeling/04_output/Fig.1.pdf]

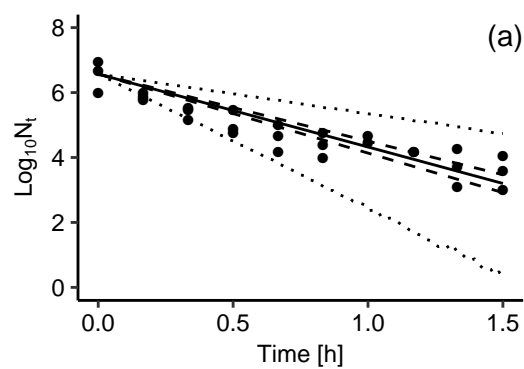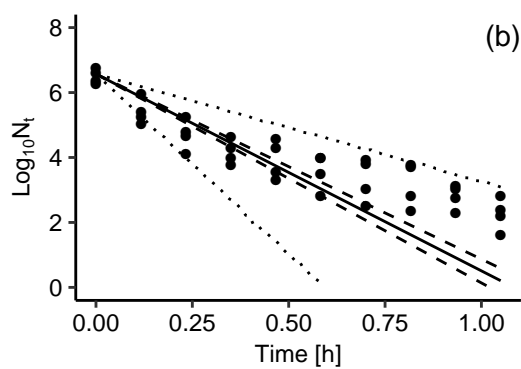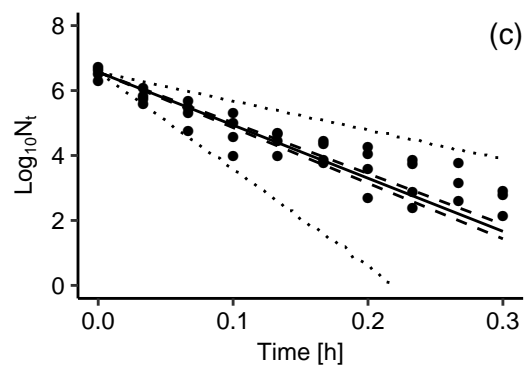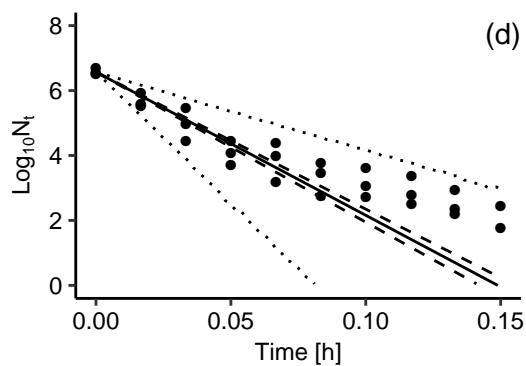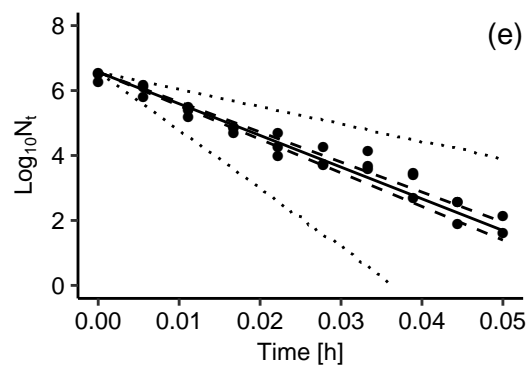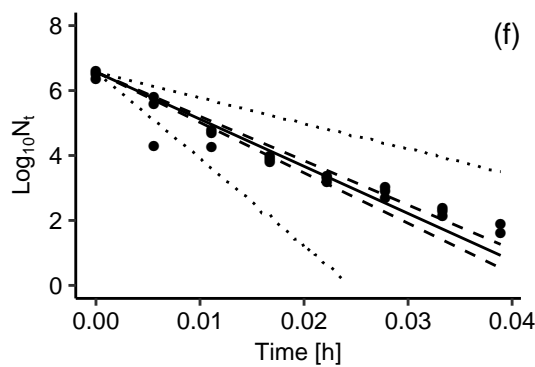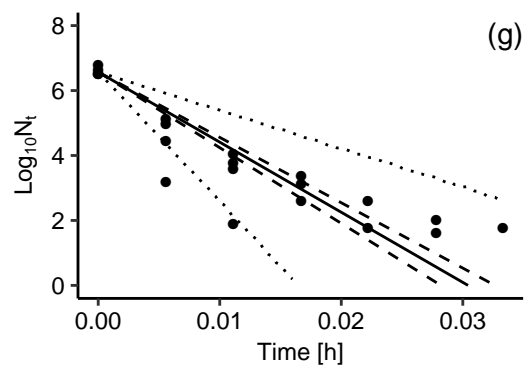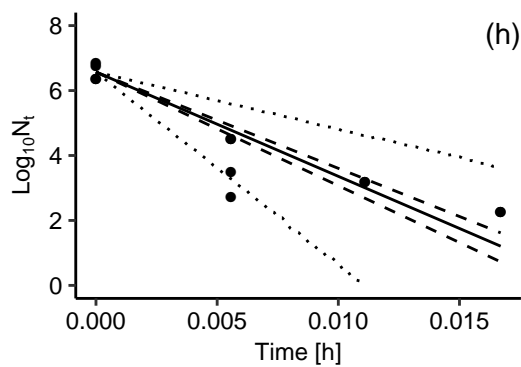

Supplement: Supplementary file 1 [file Data_Sheet_1.ZIP › bayesian_modeling/04_output/Fig.2.pdf]

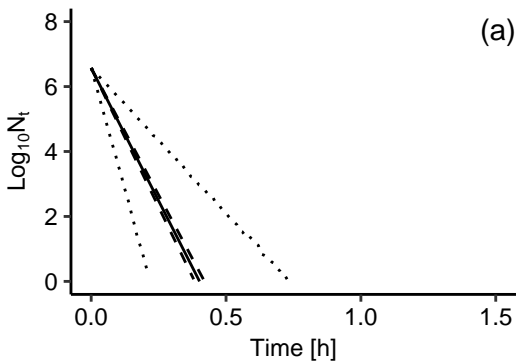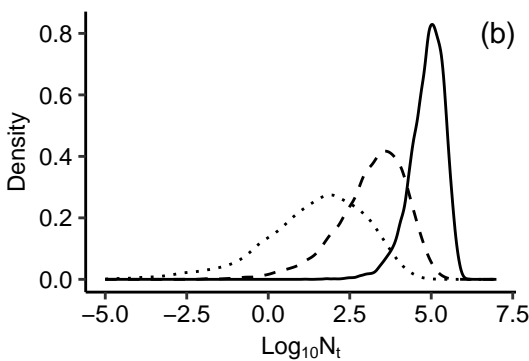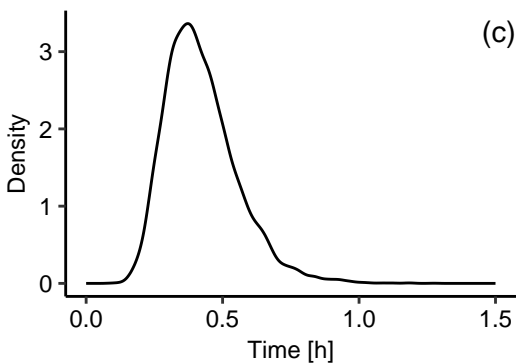

Supplement: Supplementary file 1 [file Data_Sheet_1.ZIP › bayesian_modeling/04_output/Fig.3.pdf]
